# Supplementary material for: Precision multiplexed base editing in human cells using Cas12a-derived base editors
Source: Nat Commun. 2025 May 31;16:5061. doi: 10.1038/s41467-025-59653-x (PMC12126522; doi:10.1038/s41467-025-59653-x)
Supplement: Supplementary file 1 — Supplementary Information [file 41467_2025_59653_MOESM1_ESM.pdf]

Precision multiplexed base editing in human cells using Cas12a-derived base editors

Anabel Y. Schweitzer[1,2], Etowah W. Adams[1,2], Michael T. A. Nguyen[1,2], Monkol Lek[3], Farren J. Isaacs[1,2,4,+]

[1]Department of Molecular, Cellular, and Developmental Biology, Yale University, New Haven, CT 06520, USA
[2]Systems Biology Institute, Yale University, West Haven, CT 06516, USA
[3]Department of Genetics, Yale School of Medicine, New Haven, CT, 06510, USA
[4]Department of Biomedical Engineering, Yale University, New Haven, CT 06520, USA
[+]Correspondence addressed to: FJI (farren.isaacs@yale.edu)

Supplementary Information

Supplementary Figure 1: Screening published Cas12a-derived BE systems for multiplex base editing using protocol from Wang (2020).

Supplementary Figure 2: Optimizing the multiplex base editing protocol.

Supplementary Figure 3: Screening published dAsCas12a-derived BE systems for multiplex base editing.

Supplementary Figure 4: Screening published dCas12a-derived BE systems for multiplex base editing (editing frequencies not normalized).

Supplementary Figure 5: BEACON2 mediates multiplexed base editing at RUNX1 (editing frequencies not normalized).

Supplementary Figure 6: BEACON2 mediates multiplexed base editing across 6 genes in HEK293-B2 (editing frequencies not normalized).

Supplementary Figure 7: BEACON2 mediated editing outcomes across 16 target sites in HEK293-B2.

Supplementary Figure 8: Truncated and mismatched gRNAs reduce frequencies of bystander mutations mediated by BEACON2.

Supplementary Figure 9: Screen of truncated *RUNX1* targeting gRNAs for reduced bystander mutation frequencies.

Supplementary Figure 10: BEACON1 and BEACON2 mediate multiplex base editing across multiple human cell lines (editing frequencies not normalized).

Supplementary Figure 11: BEACON1 and BEACON2 mediate multiplex base editing across 6 genes in HeLa cells (editing frequencies not normalized).

Supplementary Fig. 1

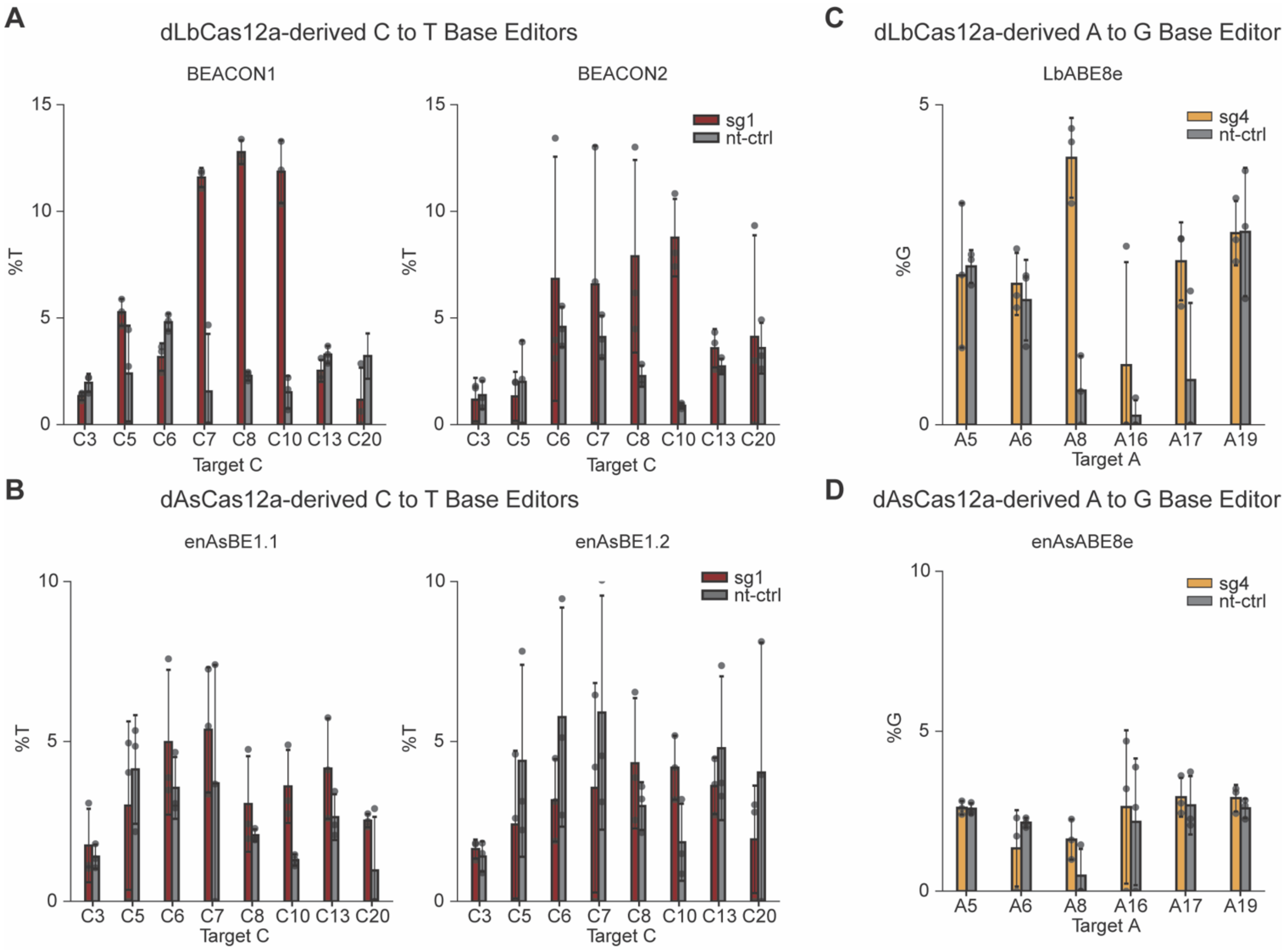

Supplementary Fig. 1: Screening published Cas12a-derived BE systems for multiplex base editing using protocol from Wang (2020). A, Comparison of two published dLbCas12a-derived CBE and **B**, two published dAsCas12a-derived CBE systems for MBE in HEK293 cells. **C**, Comparison of one published dLbCas12a-derived ABE and **D**, one published dAsLbCas12a-derived ABE for MBE in HEK293 cells. All systems were tested using a published protocol (Wang et al., 2020). sg: single guide, nt-gRNA: non-targeting gRNA. All values represent the mean±SD editing frequencies of three independent replicates (n=3). Source data are provided as a Source Data file.

Supplementary Fig. 2

A

Day	Wang et al. (2020)	Protocol 1	Protocol 2	Protocol 3	Protocol 4	Protocol 5	Protocol 6
0	seed cells						
1	transfect gRNA expression plasmid						
2	-	add DMEM with puromycin (2 µg/mL)				add DMEM with puromycin (0.5 µg/mL)	
3	-						
4	harvest	lift and replate in DMEM w/o puromycin (24-well plate)	lift and replate in DMEM w/o puromycin (12-well plate)	lift and replate in DMEM with puromycin (2 µg/mL) (24-well plate)	lift and replate in DMEM with puromycin (2 µg/mL) (12-well plate)	lift and replate in DMEM with puromycin (0.5 µg/mL) (24-well plate)	lift and replate in DMEM with puromycin (0.5 µg/mL) (12-well plate)
5		harvest	-	harvest	-	-	-
6			-		-	-	-
7			harvest		harvest	harvest	-
8							-
9							harvest

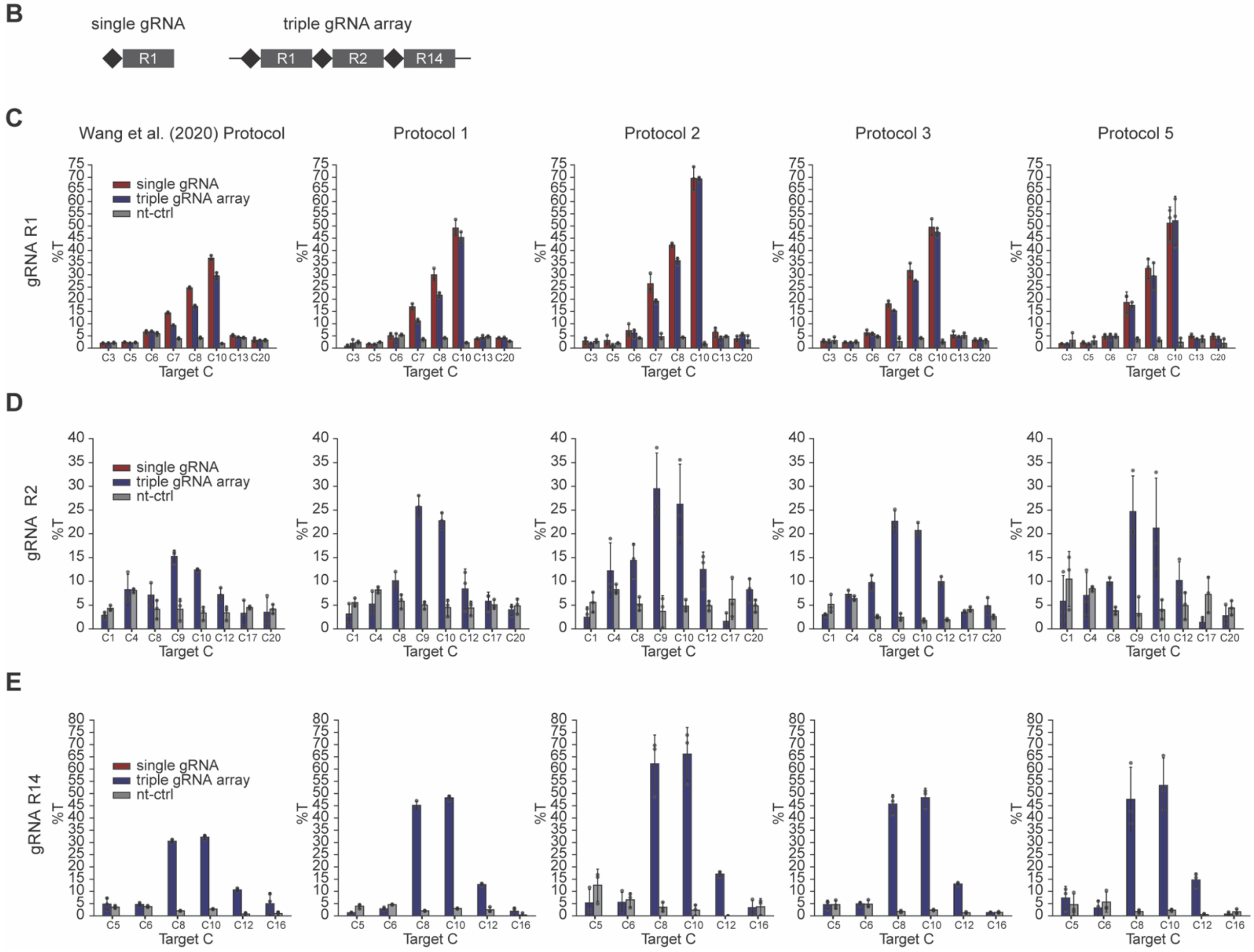

Supplementary Fig. 2: Optimizing the multiplex base editing protocol. A, List of the seven different protocols tested. Protocols for which the harvest day is marked red are protocols that did not yield enough cells for downstream processing. Protocols for which the harvest day is marked in green were processed. **B**, Schematic of the gRNA and gRNA array transfected on day 1 of the

protocols shown in A. **C**, Editing frequencies reached for the three different target sites using the tested protocols in HEK293-B2 cells. All values represent the mean±SD editing frequencies of three independent replicates (n=3). Source data are provided as a Source Data file. Statistical significance analysis of the means of each of the gRNA design categories was performed (Source Data).

Supplementary Fig.3

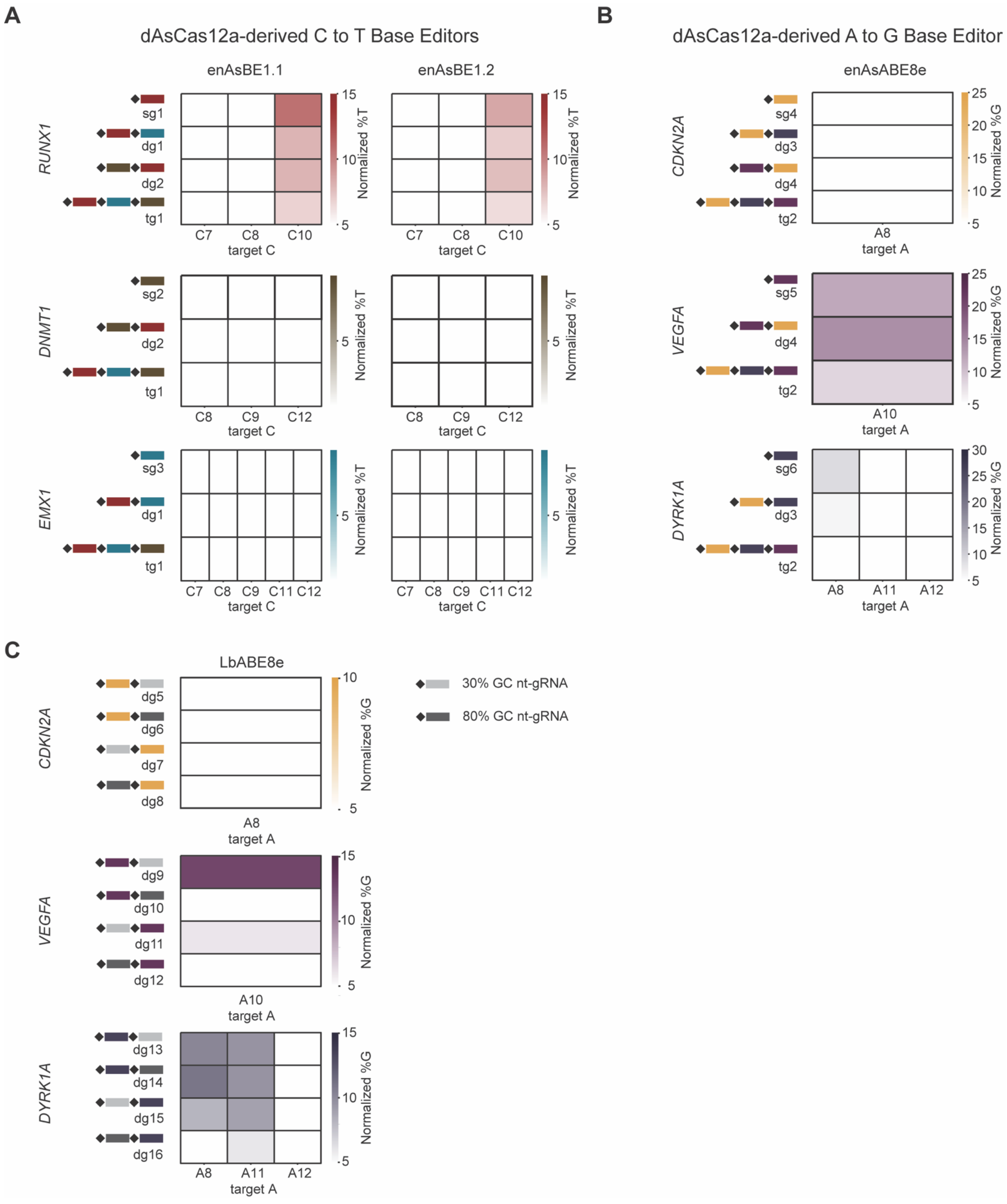

Supplementary Fig. 3: Screening published dAsCas12a-derived BE systems for multiplex base editing. **A**, Comparison of two published dAsCas12a-derived CBE and **B**, one published dAsCas12a-derived ABE systems for MBE in HEK293 cells. **C**, Editing outcomes of the LbABE8e ABE system and 12 different double gRNA arrays combining a gRNA targeting *CDKN2A, VEGFA* or *DYRK1A* with a non-targeting gRNA with 30% GC or 80% GC content. Each Heatmaps show normalized mean %T/%G values from three independent replicates (n=3). Normalization was performed by subtracting the mean %T/%G values of the nt-ctrl condition from the mean %T/%G values of the experimental condition. Only position 7-12 for CBEs and position 8-12 of ABEs are shown, as those correspond to the editing window of the used systems. sg: single guide, dg: double guide, tg: triple guide, nt-gRNA: non-targeting gRNA. Source data are provided as a Source Data file.

Supplementary Fig.4

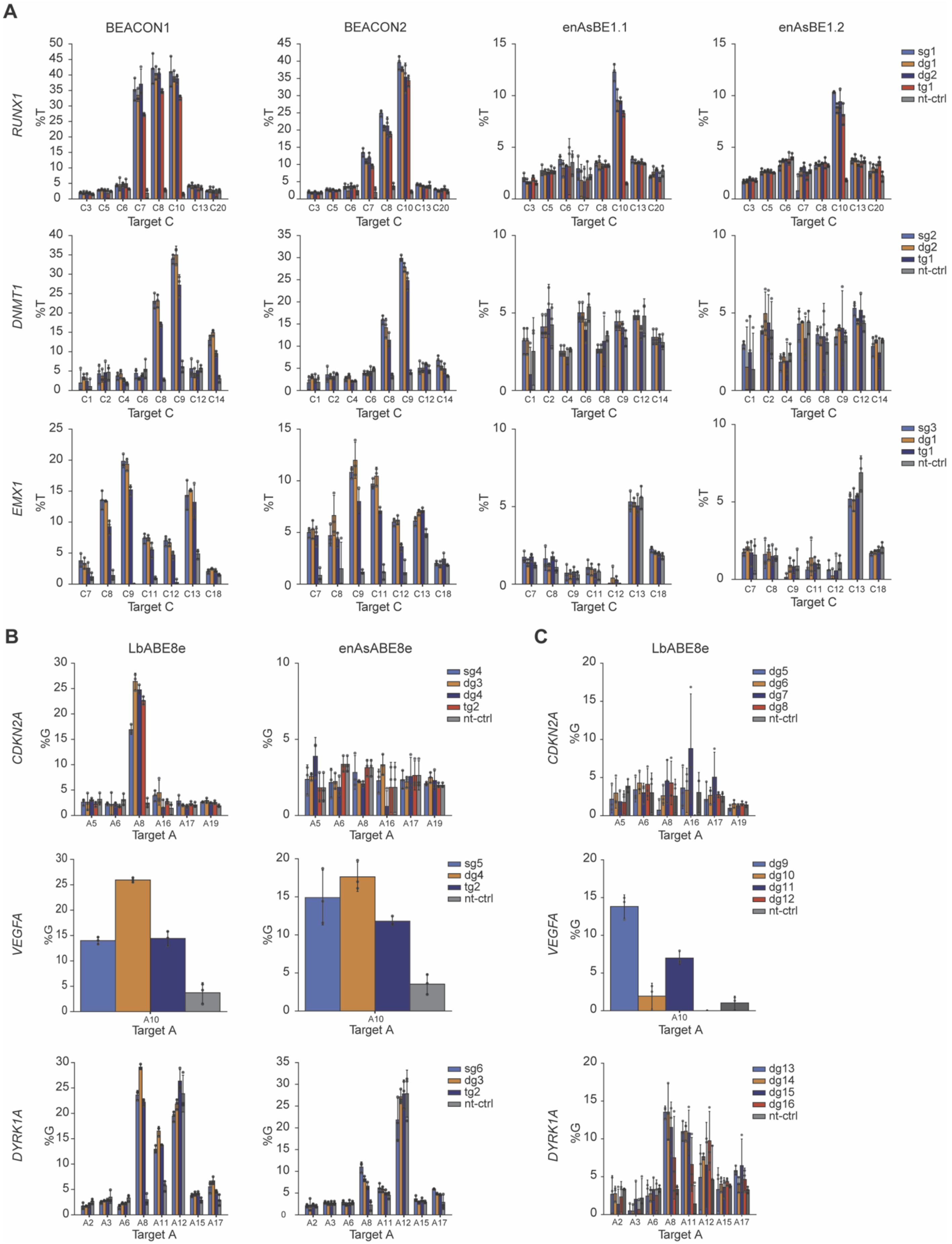

Supplementary Fig. 4: Screening published dCas12a-derived BE systems for multiplex base editing (editing frequencies not normalized). A, Comparison of four published Cas12a-derived CBE and **B**, two published Cas12a-derived ABE systems for MBE in HEK293 cells. **C**, Editing outcomes of the LbABE8e ABE system and 12 different double gRNA arrays combining a gRNA targeting *CDKN2A, VEGFA* or *DYRK1A* with a non-targeting gRNA with 30% GC or 80% GC content. All values represent the mean±SD editing frequencies of three independent replicates (n=3). sg: single guide, dg: double guide, tg: triple guide, nt-gRNA: non-targeting gRNA. Source data are provided as a Source Data file.

Supplementary Fig.5

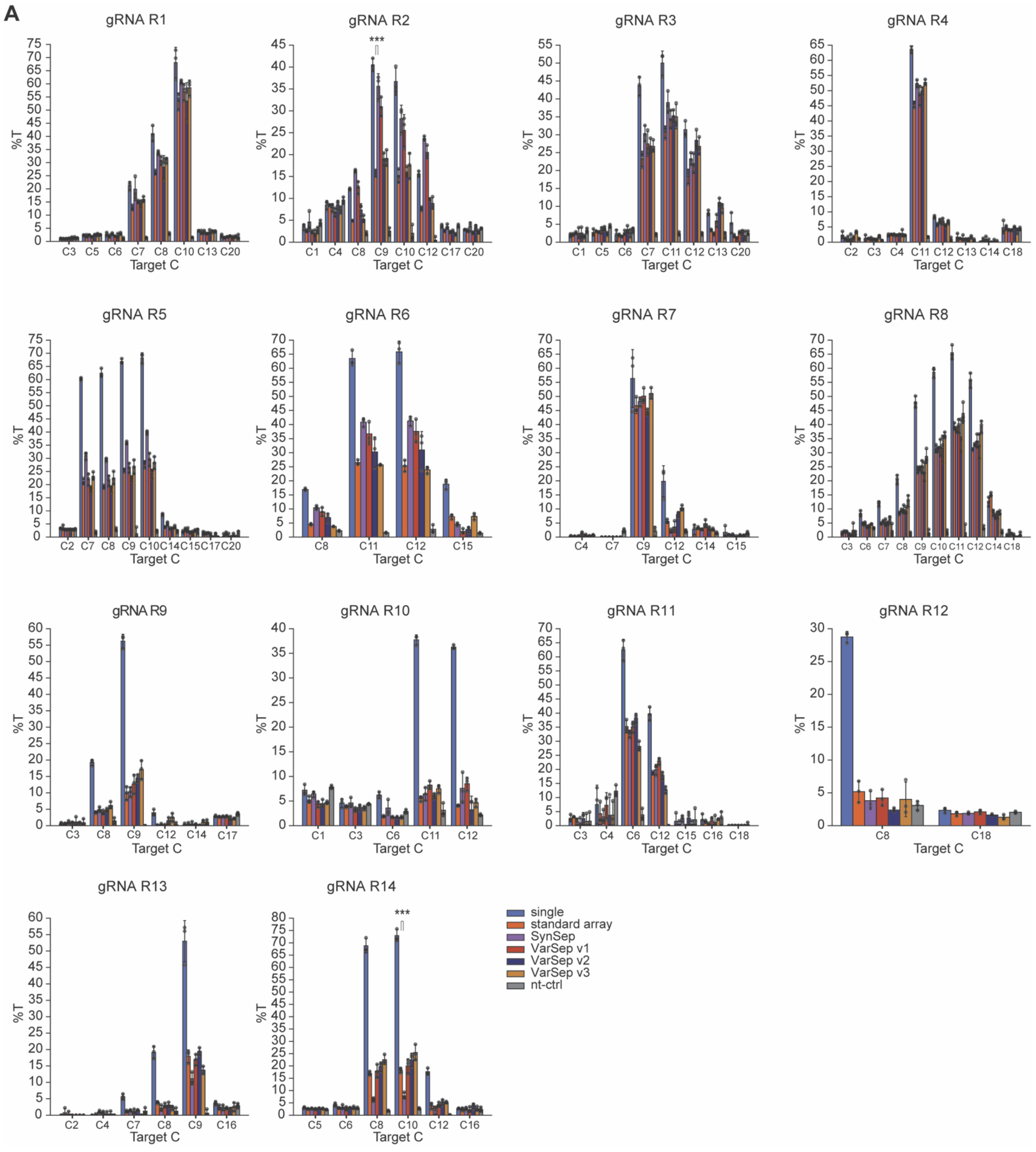

Supplementary Fig. 5: BEACON2 mediates multiplexed base editing at RUNX1 (editing frequencies not normalized). A, Editing frequencies reached for all 14 gRNAs included in the five versions of the gRNA array, as well as the gRNAs expressed as a single gRNA in HEK293-B2 cells. All values represent the mean±SD editing frequencies of three independent replicates (n=3). Source data are provided as a Source Data file. Statistical significance analysis of the means of each of the gRNA array design categories was performed (Source Data). P values from Turkey's Honest Significant Difference test for multiple comparisons. Ns, not significant ≥ 0.05, *p < 0.05 **p < 0.01, ***p < 0.001, ****p < 0.0001.frequencies

Supplementary Fig.6

A

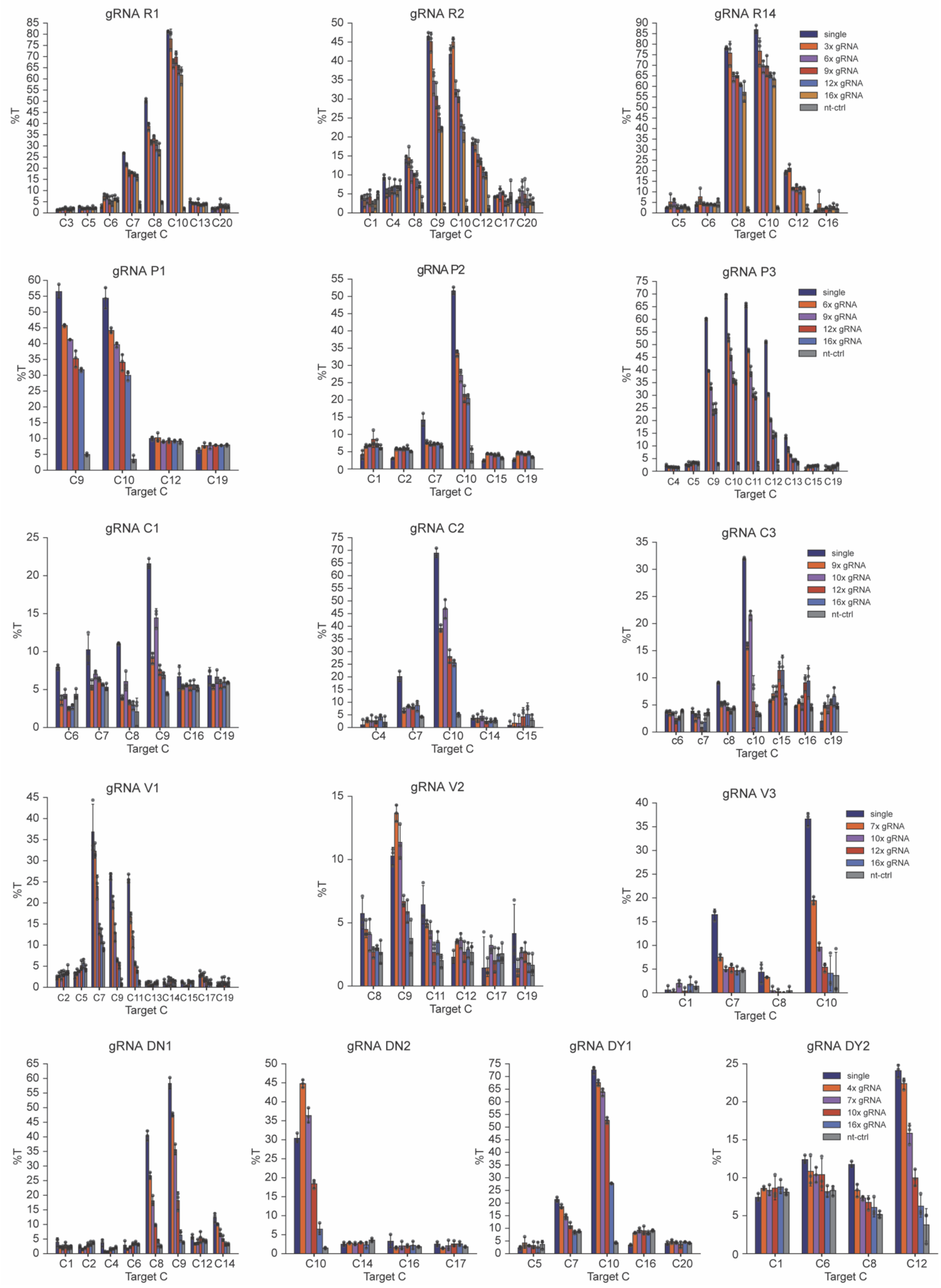

Supplementary Fig. 6: BEACON2 mediates multiplexed base editing across 6 genes in HEK293-B2 (editing frequencies not normalized). A, Editing frequencies reached for all 16 gRNAs expressed as gRNA arrays of varying length, as well as the gRNAs expressed as a single gRNA in HEK293-B2 cells. All values represent the mean±SD editing frequencies of three independent replicates (n=3). Source data are provided as a Source Data file.

Supplementary Fig.7

A

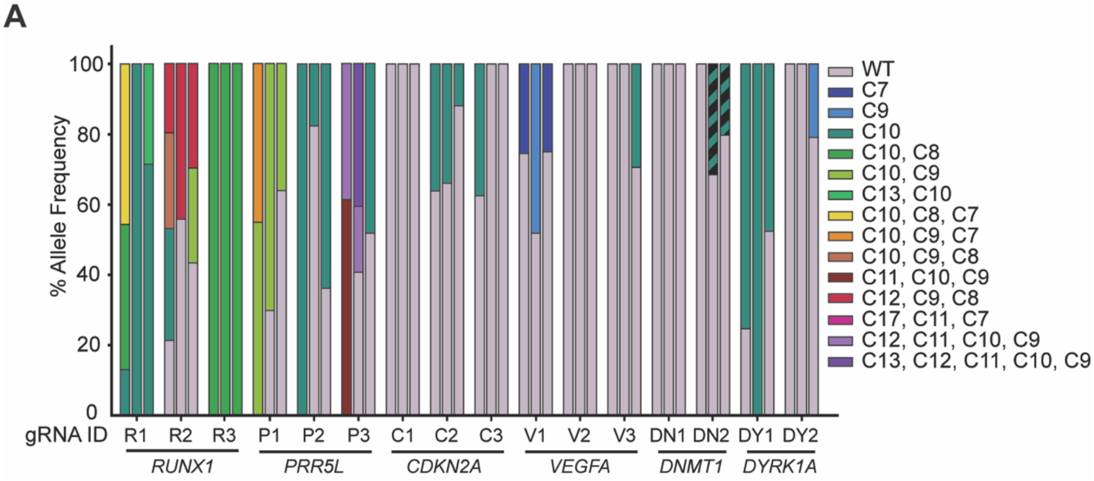

Supplementary Fig. 7: BEACON2 mediated editing outcomes across 16 target sites in HEK293-B2. A, Allele frequencies of all editing outcomes observed at the 16 targeted sites after editing with the 16x gRNA array shown in Fig. 2E. Data shown represents three cell clones isolated from the edited population. Source data are provided as a Source Data file.

Supplementary Fig.8

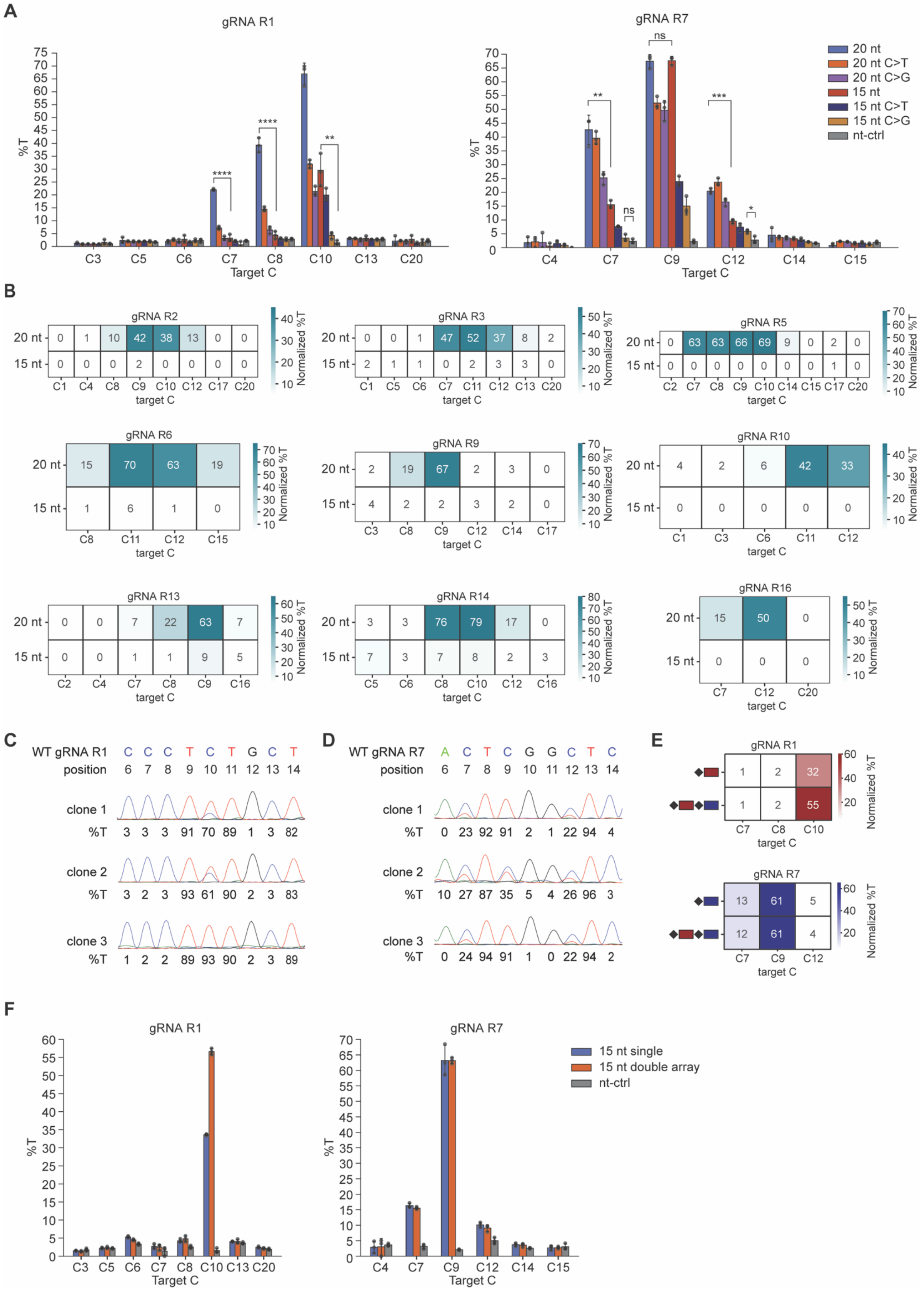

Supplementary Fig. 8: Truncated and mismatched gRNAs reduce frequencies of bystander mutations mediated by BEACON2. A, Editing outcomes of six different gRNA designs across the entire gRNA target sequence for gRNA R1 and R7. **B**, Editing outcomes of nine *RUNX1* targeting gRNAs when expressed as either individual 20 nt or individual 15 nt gRNA **C-D**, Sanger sequencing traces and %T values determined by EditR of three cell clones picked from the 15 nt wildtype sequence condition shown in A. **E-F**, Editing outcomes of 15 nt wildtype gRNAs expressed as single or double gRNA arrays. Heatmaps in F show normalized mean %T values from three independent replicates. Normalization was performed by subtracting the mean %T values of the nt-ctrl condition from the mean %T values of the experimental condition. Only position 7-12 are shown, as those correspond to the editing window of the used system. All values represent the mean±SD editing frequencies of three independent replicates (n=3). Source data are provided as a Source Data file. Statistical significance analysis of the means of each of the gRNA design categories in A was performed (Source Data). P values from Turkey's Honest Significant Difference test for multiple comparisons. Ns, not significant ≥ 0.05, $^*p < 0.05$ $^{**}p < 0.01$, $^{***}p < 0.001$, $^{****}p < 0.0001$.

Supplementary Fig.9

A

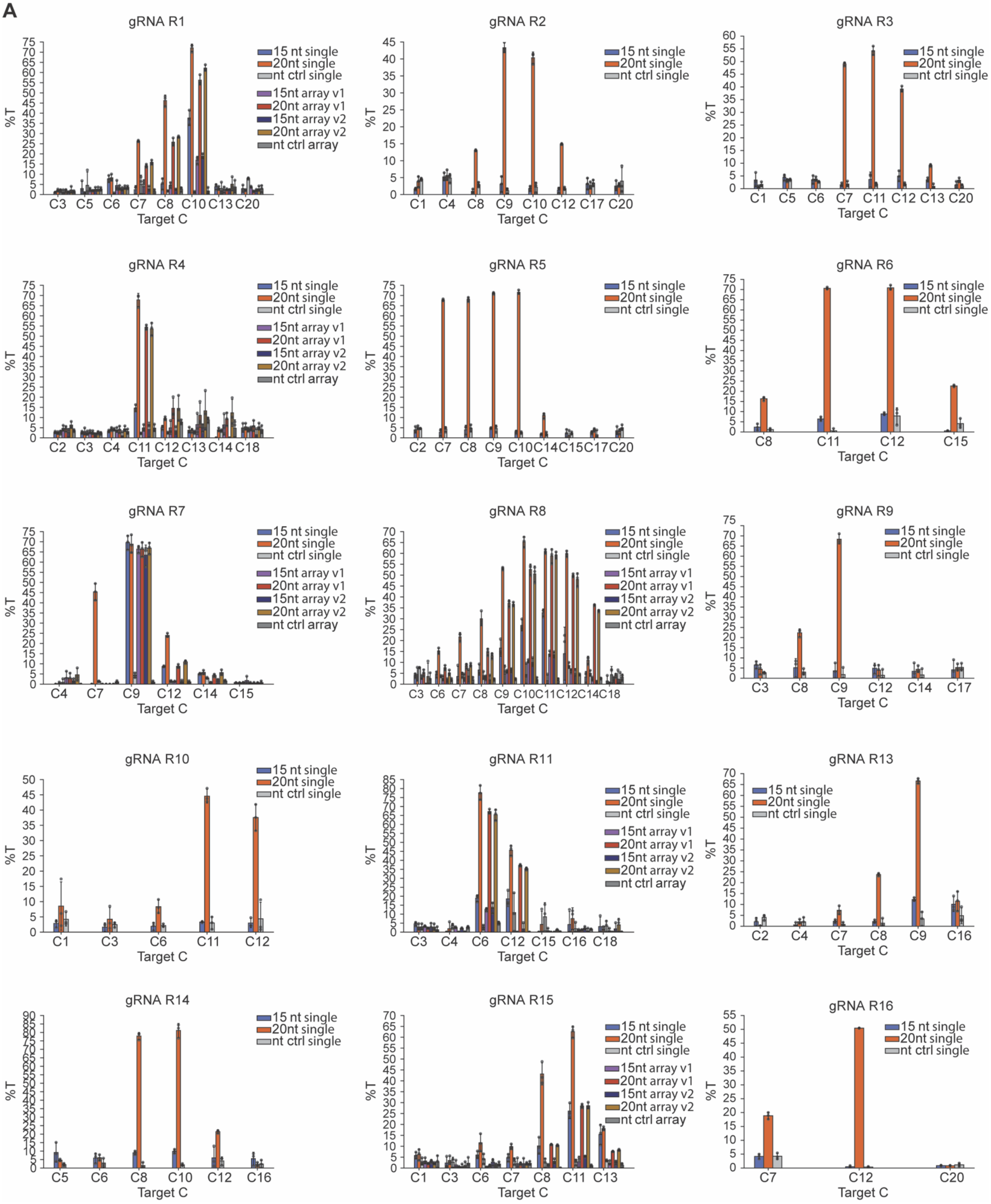

Supplementary Fig. 9: Screen of truncated *RUNX1* targeting gRNAs for reduced bystander mutation frequencies. A, Editing outcomes of 15 *RUNX1* targeting gRNAs when expressed as either individual 20 nt or individual 15 nt gRNA, or from a six gRNA array shown in Fig. 4F-G. All values represent the mean±SD editing frequencies of three independent replicates (n=3). Source data are provided as a Source Data file.

Supplementary Fig.10

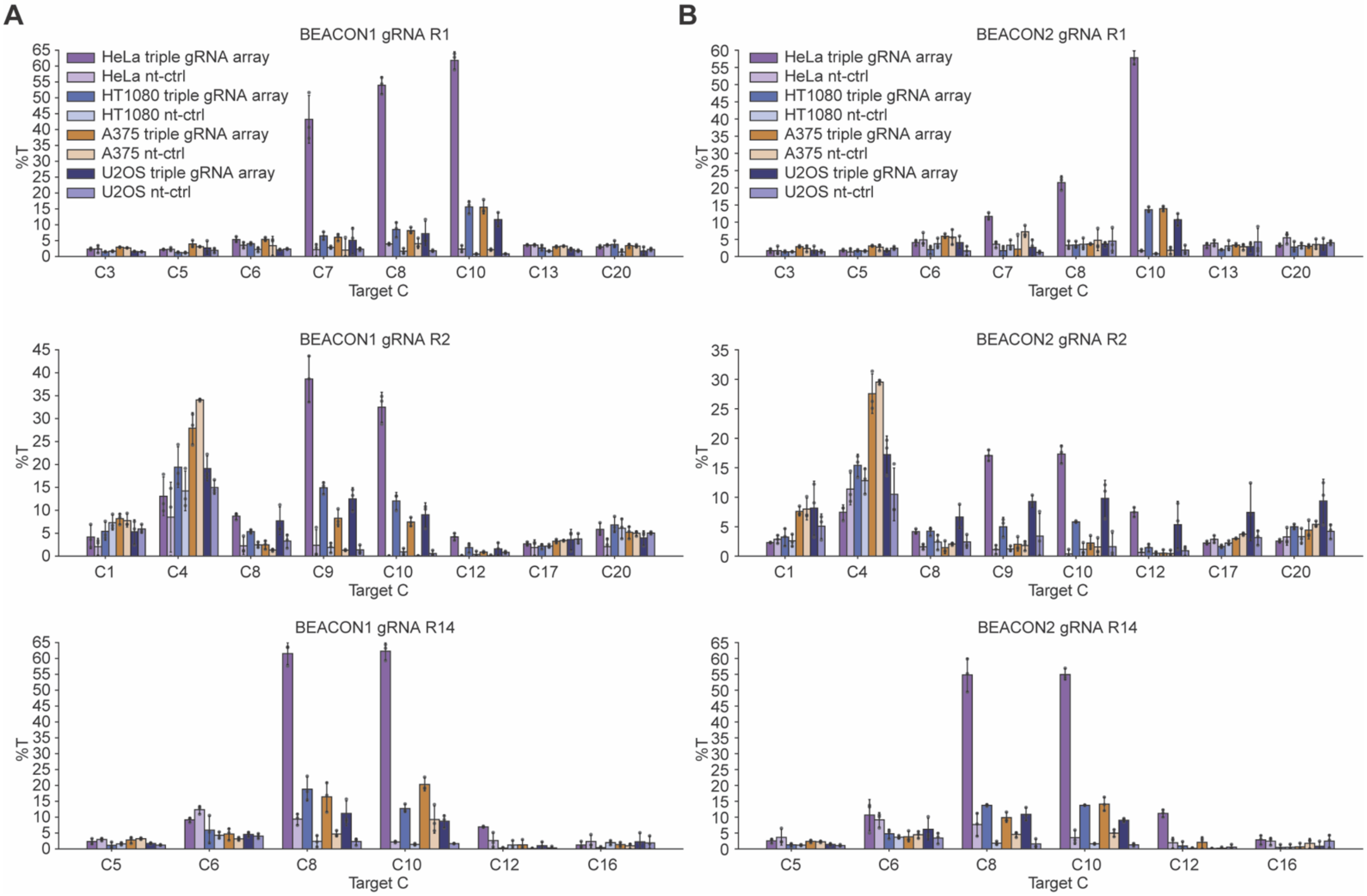

Supplementary Fig. 10: BEACON1 and BEACON2 mediate multiplex base editing across multiple human cell lines (editing frequencies not normalized). A, BEACON1-mediated and **B**, BEACON2-mediated editing frequencies with a triple gRNA array targeting *RUNX1* (Supplementary Fig. 3B) in HeLa, HT1080, A375 and U2OS cells. All values represent the mean±SD editing frequencies of three independent replicates (n=3). Source data are provided as a Source Data file.

Supplementary Fig.11

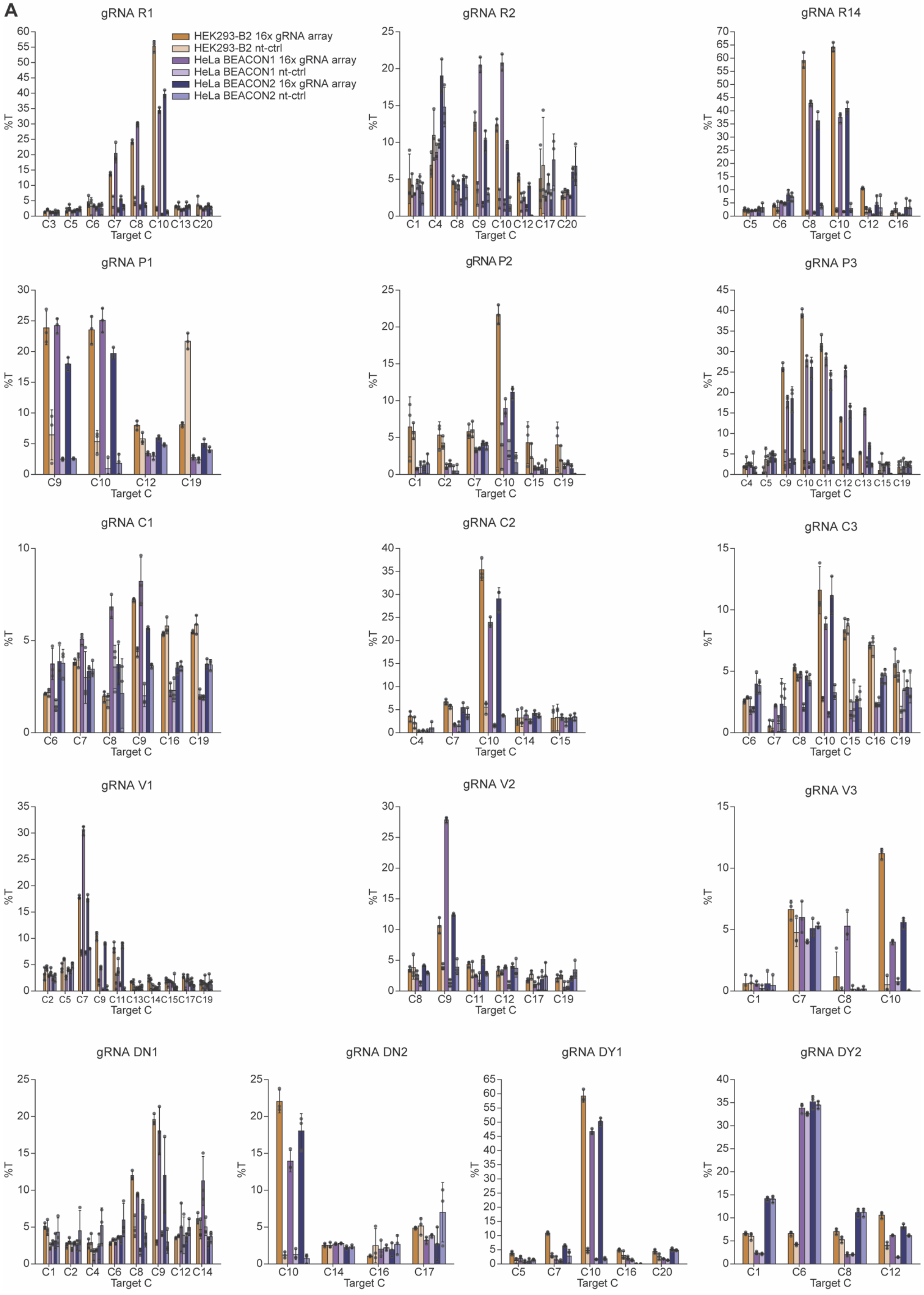

Supplementary Fig.11: BEACON1 and BEACON2 mediate multiplex base editing across 6 genes in HeLa cells (editing frequencies not normalized). A, Editing outcomes of the CMV-driven 16x gRNA array in HEK293-B2 cells and of the CMV-driven 16x gRNA array and BEACON1 or BEACON2 in HeLa cells. All values represent the mean±SD editing frequencies of three independent replicates (n=3). Source data are provided as a Source Data file.
