# Supplementary material for: Precision multiplexed base editing in human cells using Cas12a-derived base editors
Source: Nat Commun. 2025 May 31;16:5061. doi: 10.1038/s41467-025-59653-x (PMC12126522; doi:10.1038/s41467-025-59653-x)
Supplement: Supplementary file 2 — Description of Additional Supplementary Files [file 41467_2025_59653_MOESM2_ESM.pdf]

Description of Additional Supplementary Files

File Name: Supplementary Data 1
Description: This Excel workbook contains all Addgene ID numbers of plasmids used in the study, and all DNA sequences (primers, gRNAs and gRNA arrays) used in the study.
